# LGBTQ+ STEM Day 2021: an interview with Hyun Youk and Kelsey Stilson on queer experiences in STEM

**DOI:** 10.1038/s42003-021-02829-0

**Published:** 2021-11-18

## Abstract

As part of our celebration of LGBTQ+ STEM Day, we asked queer researchers and faculty about their academic experiences, role models, and how they promote an inclusive research environment.

Dr. Hyun Youk (he/him) is an Associate Professor of Systems Biology at the University of Massachusetts Chan Medical School. He moved his lab to the USA in November 2020 from the Kavli Institute of Nanoscience in Delft, the Netherlands, where he was a group leader for five years.

**Figure:**
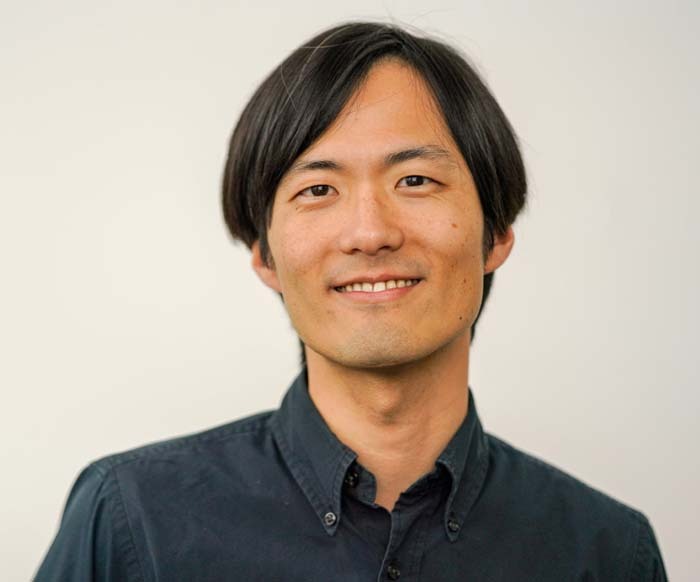
Hyun Youk.

Dr. Kelsey Stilson (they/them) is a first-year post-doctoral research fellow and anatomy instructor in the Department of Ecology, Evolution, and Organismal Biology at the Brown University Alpert School of Medicine.

**Figure:**
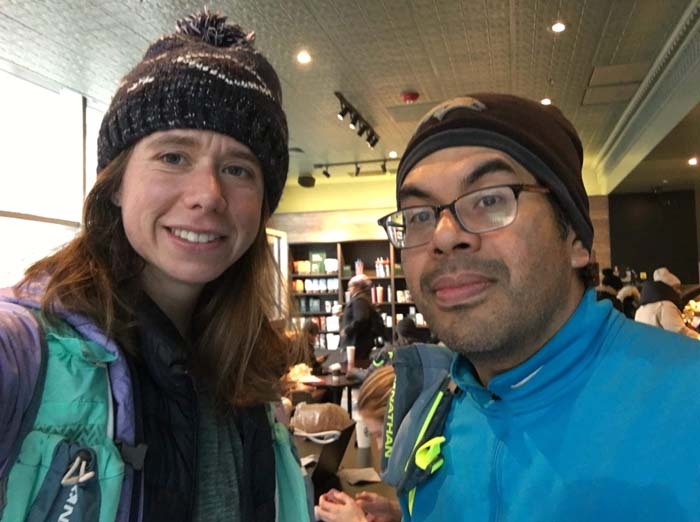
Kelsey Stilson. Kelsey Stilson (left) and their colleague, Dr. Melvin M. Bonilla (right).

Please tell me a little bit about your research interests.

**Hyun Youk (HY):** My goal in life is to deeply understand what it means for a cell or a group of cells to be alive and, in turn, what being dead really means. I’ve been fascinated with the question of why there is an (apparently) irreversible, terminal state called death and why a dynamical system “dies”. I’ve been obsessed with these questions of “life-death transition” ever since I first started learning about biology during my time as a physics PhD student. When I started in a physics PhD program, I intended to study atomic physics. But after doing a rotation in a biophysics lab of Alexander van Oudenaarden (then at MIT), I fell in love with biology and never looked back. I found biology to be difficult and fun. And I still do. The style of asking questions, answering them, and the concepts in biology all seemed so different from physics. I wanted to and still want to understand why there’s a great divide between the physical sciences (non-living systems) and biological sciences (living systems). I think a productive way to do so is by comparing a living cell to a dead cell rather than, say, to a superconductor or any other non-living system that’s far removed from a living system. I believe that we can learn a lot about life by studying cells and organisms that are dead, or very close to dying, or are in suspended life. I couldn’t pursue these topics as a trainee. I’m very fortunate to have a lab of my own that can finally pursue these topics. To make progress, my lab uses “simple” organisms like baker’s yeast. I’m very lucky to work with and learn from talented students who are now investigating how a dormant yeast spore remains alive despite looking dead and how slow life can progress without dying (the latter is by studying yeasts at freezing temperatures). Along with these benchtop experiments, we perform computer experiments (e.g., cellular automata) to understand how a handful of biological rules on a short timescale can generate complex and extremely long-lived dynamics that living systems exhibit.

**Kelsey Stilson (KS):** I have a background in paleontology, studying arthritis in rhinos and lizard skull osteology. I study how opossums masticate (chew) and teeth as a neurosensory structure. My current favorite subject is the application of Shannon-Weaver information theory to mastication systems and machine learning. I also love robots.

What does it mean to you, to be queer in STEM?

**HY:** I’m the first person in my family to attend university and there are other ways (e.g. economic means) in which I couldn’t relate to my classmates when I was a trainee. For me, being gay in academia is just another way in which I’m a bit different from many of the scientists that I meet. But frankly, I don’t think so much about being gay or different in any other way when I’m doing science. I know that I’m in this privileged position because other people, who were and are braver than I am, have fought to make academia more welcoming for people like me. Many of these are students and postdocs. One outcome of being gay—and struggling with it when I was younger like many others—is that I became more self-reflective and less scared when faced with a scientific problem or a difficulty in academia. I find that self-reflection and grit are important to survive in academia.

**KS:** Being queer in stem means being ok with ambiguity. I often have perfectionist tendencies that I apply to both my research and myself. Everything had to be “perfect” and I had to figure out the answer to every question as soon as I thought of it. So, when I realized I had questions about my identity, I would despair over what was happening inside, instead of letting the journey unfold. Just as in research, I didn’t understand that your mind needs time to absorb new concepts and feelings. You need to talk to people, learn new vocabulary, and be open to being wrong sometimes. And I didn’t understand how beautiful that journey can be when you share it with other people. I used to try to “fix” myself so I could be the “best scientist”. There are also very few statistics on the success rates of queer researchers. You can look up funding rates awarded to “men” and “women”, but what about the other genders? How are non-binary people affected by life milestones like post-docs or pregnancy in academia? How many LGBTQ+-identifying people are advisors?

My solution so far has been acceptance. Acceptance that I can’t know everything. Acceptance of, and compassion for, myself. Now I just try to figure out how to be the best Kelsey, which is not the perfect Kelsey. I practice being ok with not knowing everything right away as I explore these new frontiers. Now I understand that I can only gain understanding from experience. I learn new statistical and lab techniques as I progress with my career and who I am as a person changes over time as well. I might not be non-binary forever. I might not always work on opossums. But I am going to try my hardest to explore.

I’ve also come to accept that being queer in STEM means one is likely to have a mental health crisis. As a non-binary undergraduate and graduate student, I had a lot of issues with self-image and confidence that manifested as everything from eating disorders to anxiety attacks to self-imposed isolation. I’ve been on various medications for over half my life. I don’t tell you this to gain sympathy, but to normalize trying to survive a rigorous scientific program while society may reject you. That being said, academia also literally saved my life. The doctors working with me knew I loved science and research more than anything and would talk to me about why, scientifically, what I was doing to my body was a no-win scenario. Being queer in STEM has nothing to do with the quality of my science. In fact, scientific research and teaching has often been my refuge from myself. You don’t have to think about your inability to understand yourself and why you are constantly uncomfortable in your own body when you have work to do.

What do you think still needs to change for LGBTQ+ researchers in STEM?

**HY:** I think being in any underrepresented group is challenging. Q&A sessions like this one and the efforts by many universities and organizations to promote diversity in STEM are improving the atmosphere. For the most part, I’ve been fortunate to be in welcoming environments, like my current department and university, that are serious about diversity. But I was recently at another university where some senior academics viewed harassment and discrimination as mere perception problems on the part of the person being mistreated. There is still a lot of work to be done in academia. But I’m optimistic about the current trend.

**KS:** On a broad level, we need to rethink how we talk about sex and gender, and be comfortable with learning new vocabularies that change over time. For example, in human anatomy classes, sex and gender are often conflated. Gender and sex spectrums are framed as exceptions and donor bodies are always given genders. Yet, in paleontology we know that most fossils, out of context, can’t definitely be categorized as definitively male or female. We know that phenotype is a series of multi-dimensional spectrums. Not only are medical students implicitly taught that spectrums are exceptions and people can be put in two gender bins, but queer students themselves are alienated from the profession.

Gender bins are also very important for appearance and conference attire. The importance of “business casual” for meetings is drilled into graduate students. People that look female are expected to dress a specific way during conferences and males in another. If you dress any differently, you will be penalized. I am guilty of wearing clothing not for myself, but as a way to shape how others will treat me in a conference. You wear a costume to survive.

I am tired of being called “she”. I am tired of careful discussions where we dance around sex and gender. I am tired of having to “be one of the guys” just to talk about computers and coding. I am tired of not having a queer mentor. But I am far from the most tired person in academia. I have a lot of privilege as well. I am white and from a middle class upbringing. My parents paid for my college. I can “turn on” my friendliness and energy when I need to. I can push myself to work a long time. But it’s not the “suffer Olympics”. Queer students often beat themselves up because they feel they are too privileged to suffer. They are in the ivory tower. They have an advisor. They get to do research and work with students. Why should they be unhappy? Then I tell them, they are valid. Their suffering is valid. Their worries are valid. I am telling them so I can remind myself as well.

Who has been a role model or key mentor in STEM (LGBTQ+ or otherwise) that has had an impact on your career?

**HY:** I don’t remember knowing any professors or role models who were part of LGBTQIA+ community when I was in college and grad school. I didn’t actively search for one. During my first summer as an undergraduate student at the University of Toronto, I started doing research for the first time by working with Professor Roland List. He was in his 70s at the time and technically had “retired”. But he still came to his office nearly every day. He was also the first practicing scientist that I’ve ever met. He was using physics to model the Earth’s climate, especially cloud formation. From that summer and for the next three years, I worked on a modeling project as Roland’s sole student. He let me share his office and we worked side by side. I published one of my first papers with him. Roland taught me so much about life and research. I kept in touch with him over the years. He called me to congratulate me when my PhD work was published. He gave me a chance when no one else hired me as a research assistant. Back then, I was just looking for any summer job to pay for college. I never really thought about pursuing a research career until that summer when Roland gave me a chance. Unfortunately, he passed away two years ago. But before he passed away, I was able to tell him how much I owed him.

**KS:** My key mentor in LGBTQ+ STEM is my best friend, Dr. Melvin M. Bonilla. He is currently a staff scientist and manager of the Shubin Lab at the University of Chicago. With Melvin I can be open about my whole self, my science self, my Star Trek self, and my queer self. I am used to being the one that “gets on with it”, but I have learned the hard way that that can only lead to eventual self-destruction. Expressing myself and practicing my own vocabulary of self-acceptance with Melvin has allowed me to open up to others.

There seems to be a large gap in the sciences when it comes to queer STEM professors at R1 institutions. I personally only know one queer R1 professor in science and they are not out. Melvin and my hypothesis was that it was the Lavender Scare in the ‘50s and then the AIDS epidemic of the ‘80s and ‘90s that took our mentors away. We sometimes mourn what could have been.

How do you promote a diverse and inclusive environment within your research group?

**HY:** I don’t have any magic formula. I just try my best to be open about who I am and what I know and—most importantly—what I don’t know. I’m very open about how little I know about most subjects. I make sure that all my lab members know that I’m just as stuck as they are on their research problem. I try my best to foster an environment in which my lab members don’t feel afraid of making mistakes and trying something new. I also try to listen to my lab members rather than talk (though I don’t always succeed).

**KS:** Part of promoting an inclusive environment is to self-educate and commit to change. To really listen to everyone. To actively invite everyone into your group. To explain what you do, then listen to their interests. Give them space and time. Let them decide whether what you do is interesting to them. Direct them to another lab to try if not.

If you do field work, make sure the areas you go to are safe for a diverse range of people. If not, figure out how to make it safe. Institute a policy of no tolerance for discrimination. Have a plan if the surrounding area is hostile to people of certain ethnicities, appearances, or behaviors. Field work should not be a competition for the most able-bodied, but a collaboration that lifts everyone to the next level. People from different backgrounds will see things you never could. That can only strengthen your science and your community.

It’s also important to let people explore how they work in these new environments and let them mess up safely. Research, whether in the lab or in the field, is all about experimentation and finding a rhythm. Provide the space, then give them space to process the big ideas. Then check in. Fight perfection.

Diversity means change. It means asking for feedback on your lab and department practices, and then actually adjusting based on that feedback. It means inviting people that don’t have the same thought habits as you. That might also mean they don’t have the same work habits, eating habits, manner of dress, or cultural interests, and that’s great! If you are lucky, you will learn some things about the world.

*These interviews were conducted by Associate Editor George Inglis*.

